# Homœopathic Therapeutics in Dentistry

**Published:** 1887-07

**Authors:** 


### HOMŒOPATHIC THERAPEUTICS IN DENTISTRY.

Under this heading the editors of this journal have before had occasion (H. P., February, 1886, p. 74) to speak of a remarkable article by Dr. J. Morgan Howe, of New York, in *The Archives of Dentistry*, advocating homœopathic treatment in diseases of the teeth. In the February number (1887) of the same journal Dr. Howe contributes another article under the same heading, detailing his personal experience in such treatment. He says: "It might well be expected that cases in which functional disorders or systemic debility predominate, as exciting causes, would probably show most clearly favorable results from constitutional treatment, but such results are by no means confined to these cases. I have been in the habit of treating in this manner all cases in which it was considered advisable, without regard to the etiological diagnosis, and have not discovered that the benefits of systemic treatment are at all limited by my own ability to recognize a systemic cause for the disturbance. In my efforts to develop the benefits of systemic treatment, I have not resorted to such methods, when removal or change of local factors, mechanically or surgically, was indicated or afforded relief; but only when such means as venting by drilling, correction of malocclusion, opening abscesses, etc., were not indicated or did not afford relief."

Again, p. 53: "Tonics, anodynes, and saline cathartics I have found valuable in former years in cases requiring constitutional treatment, but I have found means adopted later much more reliable than the best results ever obtained by the former methods; remedies selected on the homœopathic basis have for several years served in my practice to control or modify pericementitis more quickly and more certainly than any other means with which I am acquainted."

The writer then proceeds to give his experience with three remedies—Mercurius Vivus, Hepar Sulphur, and Silicea. He says of Mercurius "that the period of suffering was curtailed, and suppuration—when the effort to prevent it was not successful—was less extensive and the systemic depression less marked." He has "discarded the local use of medicaments" altogether, except as *placebos*, his reliance being placed upon the mercurial preparation. So successful has he been with it that he almost regards it as a specific. The preparations used are the third and sixth decimal. Three clinical cases are given to show the value of Mercurius in pericementitis.

The indications for Hepar are given in general terms, its exact nature commented upon, and reference made to the works of Ringer, Phillips, and Wood, as being indorsements of it.

In speaking of Silicea, the worthy author seems to our mind to rather give the impression that the "common flint" is merely ground and triturated with milk sugar. For the benfit of our readers who do not know it, we may say that Silicea really passes through a stage of preparation before this is done. The quartz is first melted in a powerful furnace, with potash or soda whereby silicate of potash or soda is formed, commonly known as "water-glass," because soluble in water. This water-glass in solution is next treated with Sulphuric acid, which robs it of its alkali, and the Silicea (Silicic acid) falls in a jelly, being largely combined chemically with water, and is then triturated with sugar of milk. We are assured of the truth of this statement, as we have in our possession a letter kindly written to us by Dr. F. E. Bœricke several years since in answer to an inquiry upon this point.

To return to our author, we may say he gives us a clinical case illustrating the use of Silicea. The whole article as well as its predecessor shows such an enlightened mind that we are happy to call the attention of our readers to it, to the end that they may join us in cordial recognition and acknowledgment of his advanced position on therapeutics. W. M. J.

